# Innovative Strategies for Optimal Aging: Functional and Brain Health Research Through Community-Based Approaches

**DOI:** 10.1093/geroni/igaf122.1753

**Published:** 2025-12-31

**Authors:** Patricia Heyn, Catherine Diaz-Asper, J Taylor Harden

## Abstract

The Marymount Center for Optimal Aging (MCOA) is committed to advancing inclusive, interdisciplinary aging research that informs policy and practice. This symposium will highlight recent research initiatives in falls prevention, mental health, artificial intelligence (AI) for cognitive health, and community-based health promotion participatory research. Each presentation will discuss findings, methodological advancements, and strategies to enhance equity in aging-related interventions. Falls prevention remains a public health priority, with disparities influenced by socioeconomic status, geographic accessibility, and racial and ethnic backgrounds. Social isolation, mental health conditions, and cognitive impairment are strongly associated with falls, yet their mediating and direct relationships with fall risk remain insufficiently understood. Natural language processing (NLP) is gaining recognition as a digital biomarker for analyzing text and speech data, potentially enabling early detection and monitoring of Alzheimer’s disease. A key highlight is the Arlington Longitudinal Optimal Healthy Aging Study (ALOHA), a community-driven research initiative designed to track and improve health trajectories among diverse aging populations. ALOHA integrates fall prevention, cognitive health monitoring, and personalized wellness strategies into a scalable, interdisciplinary framework for low-funded yet high-impact aging research. Additionally, MCOA research examines challenges and solutions for supporting older adults to age in place and within their communities by exploring inequities in fall prevention programs, mental health’s role in fall risk, and AI-driven speech analysis tools as emerging biomarkers for cognitive impairment. The presentations will inform evidence-based interventions, enhance interdisciplinary approaches, and address critical gaps in aging research and community-based health promotion services.

